# Viscoelastic Properties of Porcine Pericardium Under Biaxial Tensile Creep and Stress Relaxation: Application for Novel Aortic Valve Bioprosthesis Design

**DOI:** 10.3390/bioengineering13040401

**Published:** 2026-03-30

**Authors:** Edward Matjeka, Alex G. Kuchumov, Harry M. Ngwangwa, Thanyani Pandelani, Fulufhelo Nemavhola

**Affiliations:** 1 Department of Mechanical, Bioresources, and Biomedical Engineering, University of South Africa, Pretoria 0002, South Africa; 47678534@mylife.unisa.ac.za (E.M.); ngwanhm@unisa.ac.za (H.M.N.); 2 Research Center for Genetics and Life Sciences, Sirius University of Science and Technology, Sirius 354340, Russia; kychymov@inbox.ru; 3 Biofluids Laboratory, Perm National Research Polytechnic University, Perm 614990, Russia; 4 Department of Mechanical Engineering, Faculty of Engineering and the Built Environment, Durban University of Durban, Durban 4001, South Africa; fulufhelon1@dut.ac.za

**Keywords:** biaxial testing, viscoelastic, creep, stress relaxation, porcine pericardium

## Abstract

To design novel heart valve bioprostheses, it is extremely important to predict leaflet failure and fatigue for 10–20 years, as the aortic valve opens and closes approximately 40 million times per year. Most studies devoted to aortic valve leaflets mechanical tests employ uniaxial or biaxial tests, which do not fully and explicitly describe the time-dependent biomechanical behavior of this tissue. The aim of this study was to evaluate the viscoelastic response of porcine pericardium using biaxial tensile tests. Biaxial creep tests were performed on a biaxial test machine to evaluate the circumferential and axial behavior of the porcine pericardium under creep testing, and biaxial stress relaxation was used to complement creep. The results showed that the creep behavior was the same in both directions after 1 s, 60 s, 300 s, 900 s, and 1800 s. After 30 min of creep, deformation in the circumferential and radial directions was 3303 $\times \;10^{-6}$ and 5192.9 $\times \;10^{-6}$, respectively. Stress relaxation tests showed the same behavior as creep. At stress relaxation test after 30 min, the pericardium deformation in the circumferential and radial directions was 15.28 kPa and 9.6 kPa, respectively. The Prony series with Levenberg–Marquardt as the optimizer was used to obtain material parameters to use for finite element analysis. The data obtained during such tests can be employed in numerical FSI simulations of novel aortic valve bioprosthesis long-term performance in a patient’s body.

## 1. Introduction

Aortic valve bioprostheses are widely used to replace a diseased native aortic valve to treat aortic valve stenosis (narrowing of the orifice area) [1,2,3]. Bioprosthesis leaflets are usually made based on animal tissues (ovine, bovine, or porcine pericardium is used) and specially treated for long-term performance in the human body [4,5].

Due to their resemblance to human valves, bioprosthetic aortic valves do not require anticoagulants [6]. These devices can reproduce hemodynamics close to healthy valves [7]. Nevertheless, many of these valves experience structural failure within 10–15 years after implantation due to shortcomings in the materials and design [8]. In order to attain optimal performance, enhance postoperative hemodynamics, and extend service life, these medical devices require more development. Application of new materials and coatings [9], tissue-engineered bioprostheses [10], shape and geometry optimization [11], and customized patient-specific design [12,13] are necessary to increase the efficacy of prostheses. On the other hand, in order to design novel and advanced devices for long-term performance, it is necessary to overcome the structural degeneration of bioprostheses [14,15], anisotropy and viscoelasticity of the pericardium for subsequent use in computational models [16], as well as the search and validation of alternative biomaterials [17]. Structural degeneration of the valves, often accompanied by calcification, remains a key challenge in the creation of durable bioprostheses. It was recently shown [18,19] that mechanical damage and calcification leads to catastrophically faster destruction of porcine pericardial tissue than each of them individually. A critically important conclusion is that the architecture of the tissue fiber directly determines the localization of tears and the rate of degeneration. This opens the way to pre-screening tissues based on fiber patterns to select the most stable samples, which can significantly increase the durability of prostheses. Armfield D. et al. [20] and Suárez S. et al. [21] studied the anisotropic behavior of porcine pericardium leaflets based on finite element modeling for transcatheter aortic valve replacement. The orientation of the collagen fibers in the flaps causes asymmetric deformations of the stent, which should be considered when assessing the fatigue strength of the entire device. Porcine and bovine pericardium remain the “gold standard”, but alternative sources and methods of tissue treatment are being actively explored. Rassoli et al. performed a comprehensive comparison of the mechanical properties of the pericardium of horses, pigs, donkeys, and bulls with the native valves of the human aortic valve [22]. Using relaxation tests and simulations, the authors showed that the donkey pericardium is closer to the native tissue in its characteristics (greater viscosity, lower elasticity) than pig tissue. Finite element analysis demonstrated that the valves from the donkey pericardium create less stress on the flaps, which makes it a promising candidate for new prostheses. Masoumi et al. [23] performed a comparison between treated ovine aortic valves and human native ones. Stress relaxation tests showed greater viscoelasticity in sheep tissue (41% relaxation versus 21% in human tissue over 300 s), and the FEM revealed lower peak systolic stresses. This indicates the potential advantages of sheep cloth in terms of flexibility and durability.

Creep and stress relaxation are important to evaluate how materials change when subjected to sustained stress and strain separately [24]. Viscoelastic tests are important to assess the durability of xenomaterials used to develop aortic valves. Creep and stress relaxation tests are essential in helping us predict the life expectancy of an implanted aortic valve and avoid any reintervention, decreasing the patient’s life quality. To our knowledge, creep and stress relaxation tests for the porcine pericardium are limited [22,25,26,27,28], and this study was carried out to address the behavior of the pericardium under creep and stress relaxation.

Properties of the native porcine pericardium material are important in the development of desirable bioprosthetic aortic valves; the viscoelastic properties of the porcine pericardium are not well addressed in the literature. Most viscoelastic tests carried out by other researchers are uniaxial for the pericardium or biaxial for leaflets of the aortic valves and mitral valves [22,29]. Viscoelastic properties are important to provide comprehensive material parameters of the porcine pericardium [30]. The aim of this study was to find porcine pericardium viscoelastic parameters by using biaxial tensile tests. Creep tests were performed on a biaxial test machine to evaluate circumferential and radial responses of the pericardium under creep testing; biaxial stress relaxation was used to complement creep in finding viscoelastic material parameters of porcine pericardium. Prony series models were used to find viscoelastic material parameters, the generalized Kelvin–Voigt model was used for creep test predictions, and the generalized Maxwell model was used to predict stress relaxation tests. To optimize the material parameters obtained, the Levenberg–Marquardt optimizer was used. Authors expect that this study can help to improve the design of transcatheter aortic valve intervention leaflets, predict valve tears, and make progress with low-gradient stenosis in some patients. Moreover, the data obtained during such tests can be employed in numerical FSI simulations of novel aortic valve bioprosthesis long-term performance in a patient’s body.

## 2. Materials and Methods

### 2.1. Tissue Acquisition

**Figure 1:**
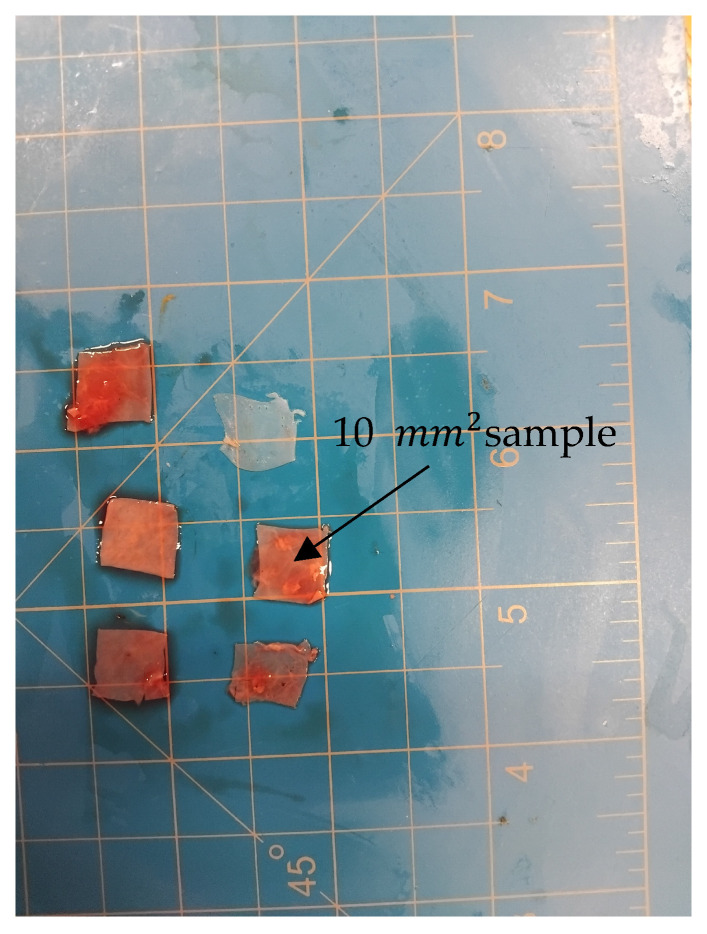
Landrace porcine pericardium samples dissected into sizes of 10 ${mm}^{2}$ for viscoelastic testing.

Porcine hearts were collected from a local abattoir using a cooler box with ice; collection process took place immediately after the slaughtering of 51-week-old pigs. Samples were taken to the laboratory, which is 15 min from the laboratory. Upon arrival, each heart’s pericardium was excised to 10 ${mm}^{2}$. Samples for testing are shown in Figure 1.

Figure 1 shows some porcine pericardium which was used for viscoelastic tests; samples were placed on top of a cutting board, which was used to measure samples and cutting.

### 2.2. Experimental Equipment

#### Cellscale Biotester 5000

**Figure 2:**
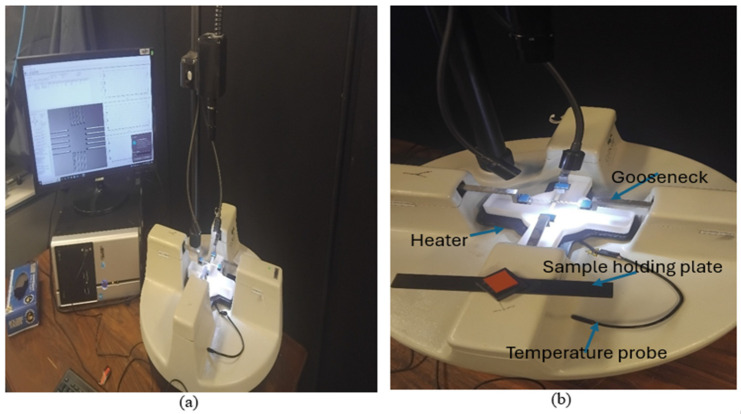
(**a**) Cellscale Biotester 5000 system, (**b**) Goosenecks, temperature probe, samples mounting plate and heater.

**Figure 3:**
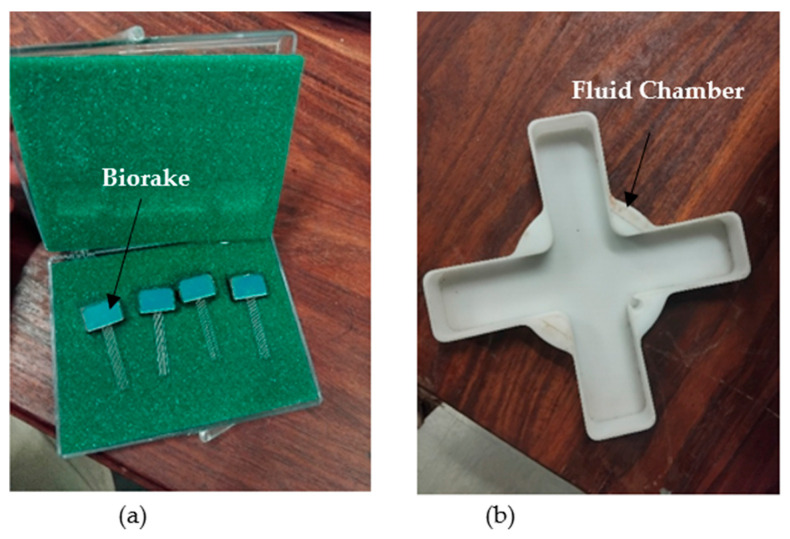
(**a**) Samples mounting biorakes for biaxial testing, (**b**) fluid chamber to pour PBS.

For biaxial creep and stress relaxation tests, the Cellscale biaxial biotester machine (Waterloo, ON, Canada) was used [31]. The Cellscale biaxial biotester machine setup is shown in Figure 2. The biotester has four goosenecks mounted with 23 N load cells on each of them, and moving in the opposite direction to apply tension to the mounted samples. The sample-holding equipment used on the biotester is the biorakes shown in Figure 3a. To calibrate the biotester, calibration loads were used on both axes, and load vs. displacement readings were taken using the software that comes with the biotester. The temperature-reading probe was calibrated by pouring water into the fluid chamber and setting the temperature to 37 °C on the software. When the probe was not submerged in the fluid chamber, when the probe was outside the chamber, the readings on the software were approximately 25 °C and 37 °C once placed inside the fluid chamber, shown in Figure 3b. To calculate stress from the obtained force, the following equations were used.

(1) $$\sigma =\frac{f_{n}}{A}$$

(2) A=Lt

where A is the area denoted by the product of length L and thickness t, $f_{n}$ is the maximum applied force.

### 2.3. Biaxial Creep Testing

Porcine pericardium of 10 ${mm}^{2}$ was mounted to a Cellscale biotester with PBS in the fluid chamber, and the temperature was raised to 37 °C to mimic physiological conditions [32]. To avoid hysteresis, a preload of 0.1 N was applied at the start of the preconditioning process [33]. To bring samples to their operating conditions, preconditioning was applied [34] and dissected into two parts, the first part consisting of 5 preconditioning cycles loading to 1 N for 20 s and unloading for 20 s, the second preconditioning stage consisted of 10 cycles, with the only difference from the first stage being to hold for 90 s every time a maximum force of 1 N is reached. Creep tests were also performed by applying a maximum force of 1 N for a duration of 30 min. This was performed at equibiaxial tensile loading using biorakes to grip the specimen in the circumferential and radial directions.

### 2.4. Biaxial Stress Relaxation

Subjecting a specimen to constant strain to evaluate its change in stress is called stress relaxation [35], which is one of the important methods to effectively characterize a material. The stress relaxation test carried out in this study, preconditioning setups, is similar to that described in creep tests. The only difference being that in stress relaxation, we replaced the maximum applied force with a 10% strain.

### 2.5. Viscoelastic Testing Analysis

#### 2.5.1. Biaxial Tensile Creep Analysis

The biaxial tensile creep test produced deformation versus time in the axial and circumferential directions. The deformation was divided by the original length to get the strains and strains at times of 1 s, 60 s, 300 s, 900 s, and 1800 s, which were statistically compared for seven creep experiments between two directions using one-way ANOVA. To obtain viscoelastic material parameters, the generalized Kelvin–Voigt model was used for both directions [36]; the optimizer used was the Levenberg–Marquardt optimizer [37], and the model function is written as follows.

(3) $$J\left(t\right)=J_{0}+\sum_{i=1}^{N}J_{i}(1-e^{-\frac{t}{\tau_{i}}})$$

where $J_{0}$ is the instantaneous elastic compliance, $J_{i}$ is the compliance coefficient, $\tau_{i}$ is the retardation time, and t is the time. The strain prediction correlation is denoted as follows.

(4) $$\varepsilon \left(t\right)=J(t)\sigma_{0}$$

#### 2.5.2. Biaxial Tensile Stress Relaxation Analysis

The stress relaxation test gave us force versus time in the radial and circumferential directions, and the force was divided by the area to get the stresses in both directions. Statistical comparisons of stress relaxation in both directions were made at times of 1 s, 60 s, 300 s, 900 s, and 1800 s by using one-way ANOVA. The Levenberg–Marquardt optimizer was used to obtain material parameters from the generalized Maxwell model [38], which is written as follows.

(5) $$E\left(t\right)=E_{0}+\sum_{i=1}^{N}E_{i}(e^{-\frac{t}{\tau_{i}}})$$

where $E_{0}$ is the instantaneous elastic modulus, $E_{i}$ are the modulus of the springs. The predicted stress as a function of time is denoted as follows.

(6) $$\sigma \left(t\right)=E(t)\varepsilon_{0}$$

#### 2.5.3. Error Terms

To measure the goodness of fit, the absolute sum of errors (ASEs), coefficient of determination (R2), coefficient of correlation (r), and the normalized root mean square error (NRMSE) were used.

#### 2.5.4. Statistical Analysis

The circumferential and radial responses of porcine pericardium were compared at different times for both creep and relaxation tests using one-way ANOVA with the assumption of homogeneity of variance.

## 3. Results

### 3.1. Viscoelastic Tests (Creep and Stress Relaxation)

#### 3.1.1. Biaxial Creep Tests

**Figure 4:**
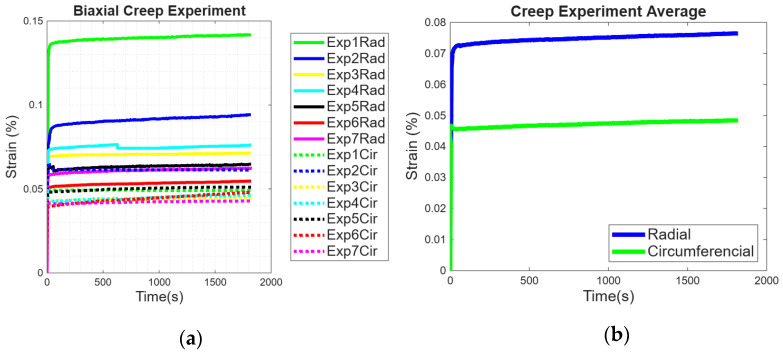
(**a**) Creep test results in the circumferential and radial direction of the pericardium, (**b**) experimental results averages.

For biaxial creep, the applied force was sustained at 0.5 N for 30 min, and strain changes in the circumferential and radial directions were evaluated. The anisotropic behavior of the pericardium can be observed from all seven experiments shown in Figure 4a. At first, the strain applied in the circumferential direction started by decreasing for most samples, indicating Poisson’s effects, and later increasing in all directions, with the radial direction being more compliant. Both directions were seen to have a minimal increase in strains on average, which was less than 1%, and this was seen in Figure 4b.

#### 3.1.2. Biaxial Stress Relaxation

**Figure 5:**
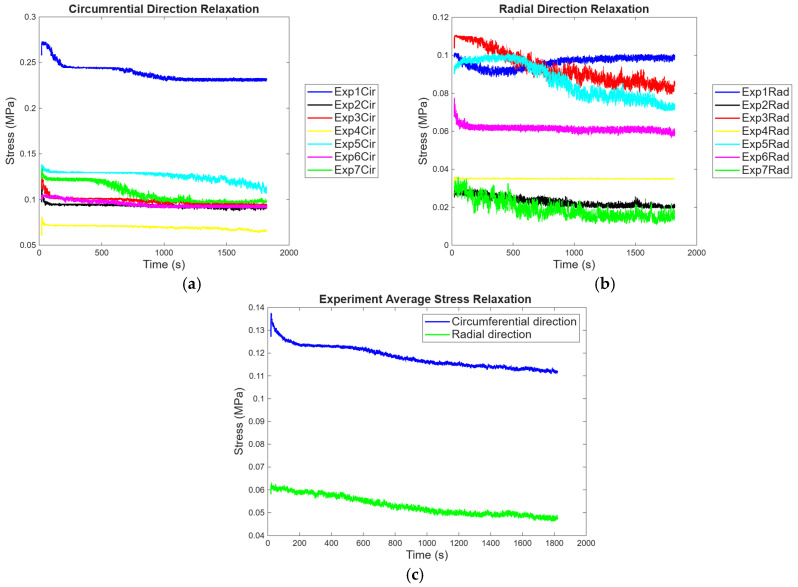
(**a**) Stress relaxation for 7 experiments in the circumferential direction, (**b**) stress relaxation in the radial direction, and (**c**) both direction’s averaged result.

The biaxial stress relaxation test was carried out to evaluate the time-dependent behavior of the porcine pericardium in both the circumferential and the radial direction to mimic the multiaxial loading that occurs under physiological conditions. When comparing stress relaxations in the circumferential and radial directions, five-time intervals were used; the intervals used are 1 s, 60 s, 300 s, 900 s, and 1800 s. Figure 5a,b show seven experimental results after performing biaxial stress relaxation to obtain the responses in the circumferential and radial directions of the pericardium. For all experiments, the circumferential direction was taking more stress than the radial direction. After taking the average of all experiments and plotting the results as depicted in Figure 5c, stress relaxation for seven experiments and their average result, both the circumferential and the radial directions relaxed at the same rate, and the circumferential direction was seen to be carrying 0.13 MPa initially and decreased to 0.11 MPa after 30 min, whereas the radial direction’s initial stress was at 0.06 MPa and relaxed to 0.045 MPa.

### 3.2. Viscoelastic Material Parameters

#### Generalized Kelvin–Voigt Viscoelastic Model Used for Creep Test Results, and Generalized Maxwell Model Used for Stress Relaxation

**Figure 6:**
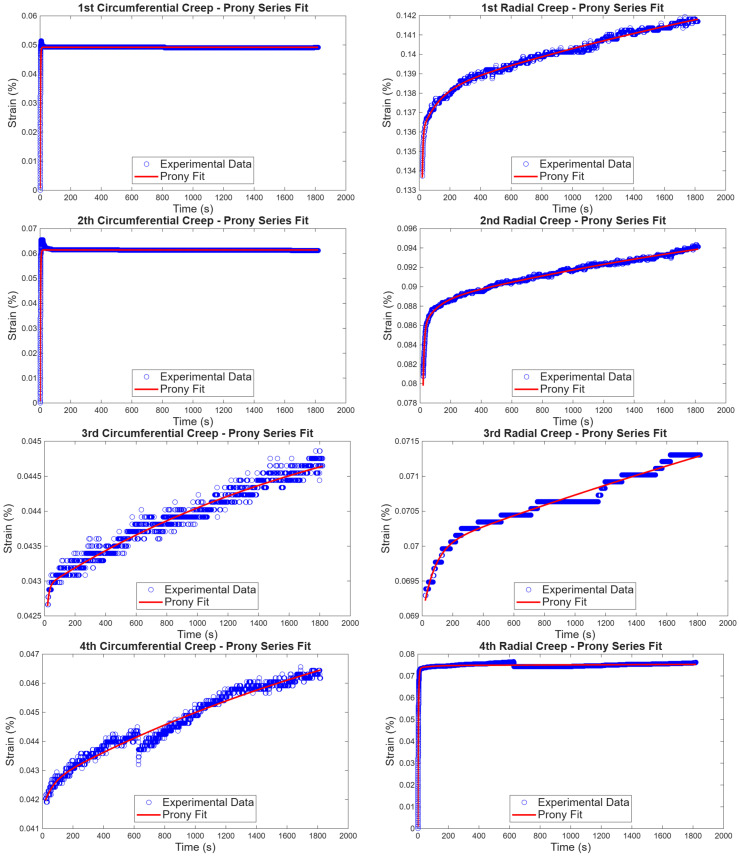

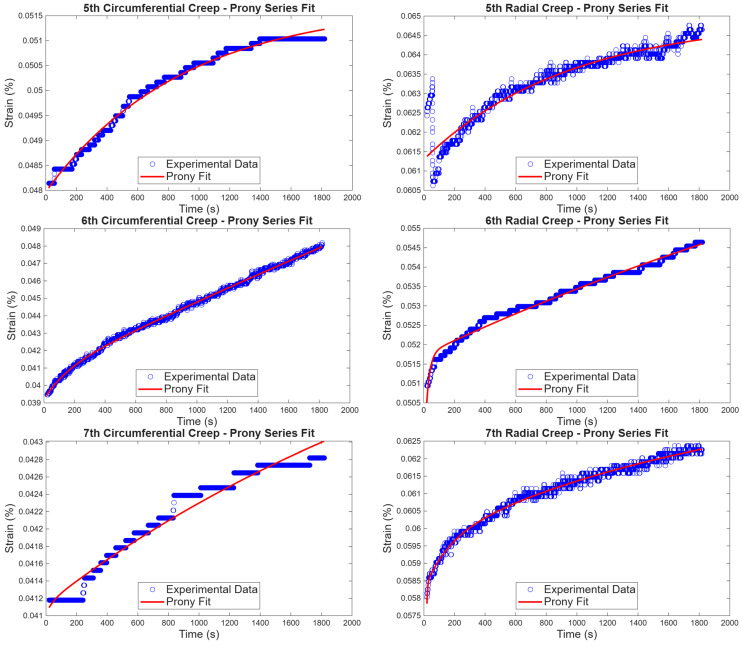
Generalized Kelvin–Voigt fitting from creep experimental results in the circumferential and radial directions.

**Figure 7:**
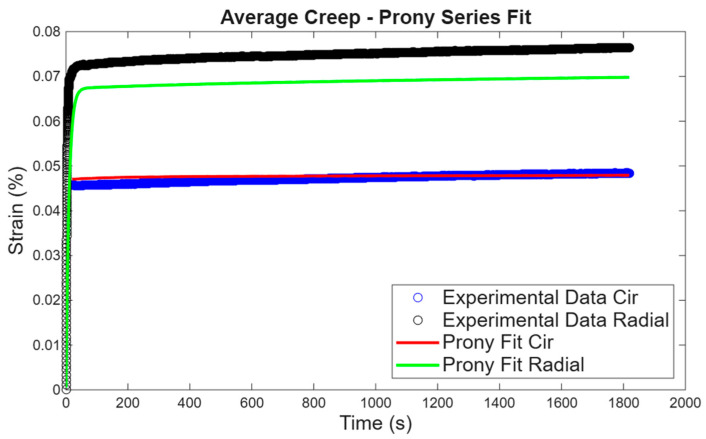
Average parameters fitted from average experimental data in both directions.

The material parameters for viscoelastic constitutive models, which are the generalized Kelvin–Voigt and the generalized Maxwell models, are shown in Table 1, Table 2, Table 3 and Table 4 below. The generalized Kelvin–Voigt parameters for the circumferential direction and radial direction are shown in Table 1 and Table 2, respectively. The average coefficient of determination obtained for material parameters in the circumferential direction was 0.96 and 0.98 for the radial direction, which indicates desirable results. The generalized Maxwell parameters in the circumferential direction and radial direction are shown in Table 3 and Table 4, respectively. Material parameters for the circumferential direction were found with the coefficient of determination of 0.89 and 0.75 in the radial direction. For the creep test, the strain vs. time plots of the experimental data and generalized predictions of the Kelvin–Voigt model are shown in Figure 6. The initial material parameters used were guessed for this model. The obtained material averages were used to predict the average experimental data in both directions, and the strain vs. time graph is shown in Figure 7. The first and second experimental reactions of the strains in the circumferential direction started by decreasing, which led us to fit the model to the entire experimental data from zero stress to maximum stress.

The Kelvin–Voigt model fitting to creep results for circumferential and radial directions is given in Figure 6. The coefficients of determination were above 96% for both directions.

The overall performance of the model was taken by taking the average of experimental results and using average material properties to fit the average experimental results, which showed good results, as shown in Figure 7.

**Figure 8:**
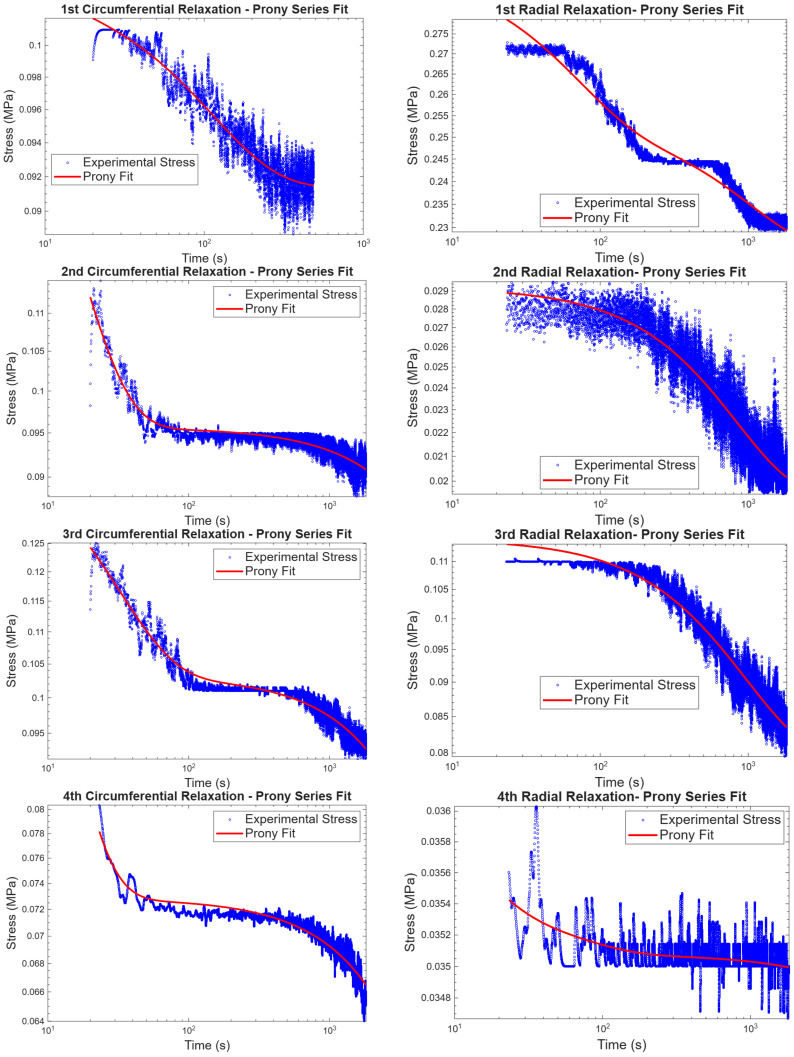

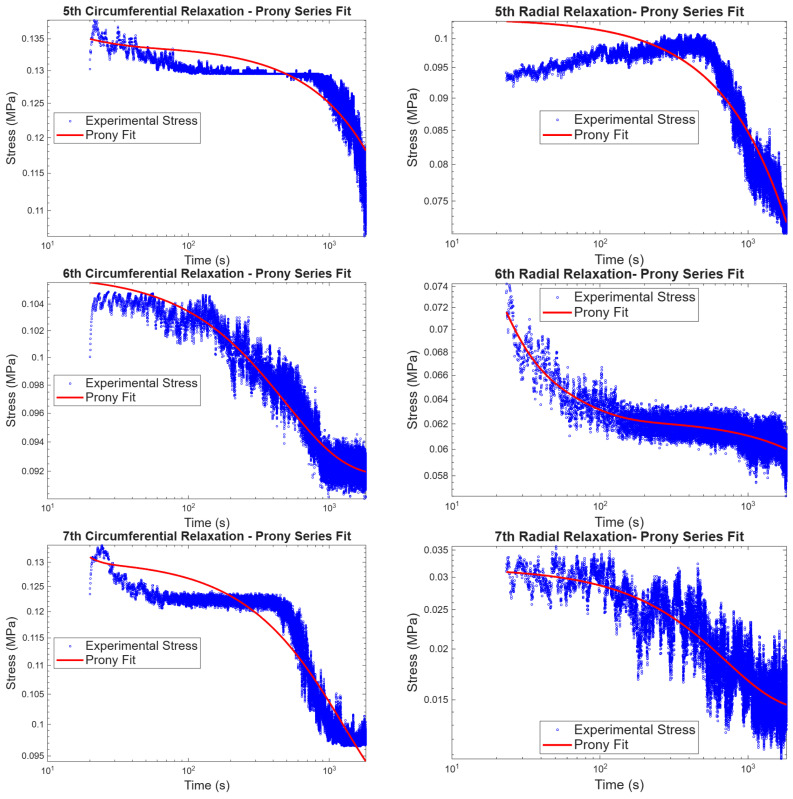
Generalized Maxwell model fitting from creep experimental results in the circumferential and radial directions.

**Figure 9:**
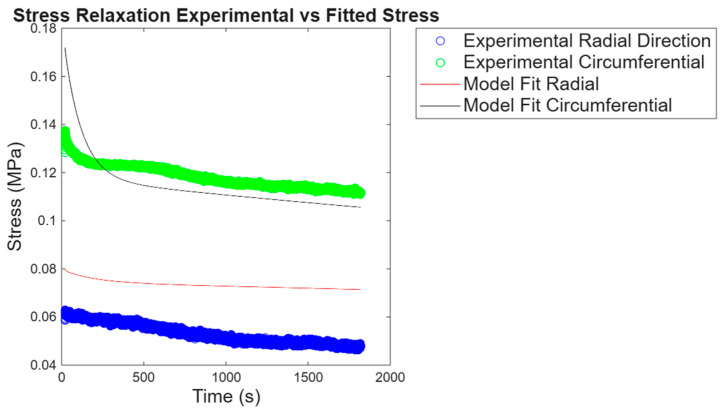
Generalized Maxwell model average parameters fitted to average experimental data in both directions.

Generalized Maxwell model (three-term Prony series) used for stress relaxation. The generalized Maxwell model was used to predict experimental stress relaxation, and the fit of the model to experimental data in the circumferential and radial directions is shown in Figure 8. To initialize material parameters, the initial stress at 0.5 N was used as $\sigma_{0}$ and a different percentage of $\sigma_{0}$ to define $\sigma_{i}$. The retardation times were chosen arbitrarily. Taking the average material parameters obtained and fitting them to the average experimental results, the parameters proved to be reliable, as shown in Figure 9.

### 3.3. Statistical Analysis

#### 3.3.1. Creep Test Statistical Analysis

Homogeneity of variance was an assumption used when using one-way ANOVA. Since the initial creep strains are different in all circumferential and radial directions, strain differences at 1 s, 60 s, 300 s, 900 s, and 1800 s were compared for the responses in the circumferential and radial directions. After 1 s, the change in deformation in the circumferential direction was 27 ± 70.5 $\times \;10^{-6}$, which showed no statistical significance compared to the radial direction 163.95 ± 250 $\times \;10^{-6}$ with (*p* = 0.1868). For all remaining chosen time intervals, strain changes in the circumferential and radial directions had no significant differences, where for 60 s in the circumferential direction 727.7 ± 1020.8 $\times \;10^{-6}$ and radial direction 2140 ± 2376.7 $\times \;10^{-6}$ with (*p* = 0.1742). After 300 s the circumferential strain was 1264.3 ± 1056.7 $\times \;10^{-6}$, in the radial direction 2871.6 ± 2968.9 $\times \;10^{-6}$ where (*p* = 0.2021). The 15 min interval is considered by other authors as the time at which a large strain occurs [18], and no significant difference was observed in the circumferential direction 2277.5 ± 1507.6 $\times \;10^{-6}$, and the radial creep was 3791 ± 3672 (*p* = 0.333). After the whole creep test interval, no significant differences were observed in the circumferential direction 3303 ± 2648.7 $\times \;10^{-6}$ and the radial direction 5192.9 ± 4133.7 $\times \;10^{-6}$ (*p* = 0.3285).

#### 3.3.2. Stress Relaxation Statistical Analysis

Homogeneity of variance was used as an assumption for one-way ANOVA and verified. After 1 s, there was statistical significance between the circumferential and radial direction 10.02 ± 3.59 kPa and 3.82 ± 2.43 kPa, respectively (*p* = 0.0026). After 1 min of stress relaxation, no statistical significance was observed in the circumferential 4.63 ± 4.29 kPa and the radial directions 3.48 ± 1.73 kPa (*p* = 0.5224). The same can be said about relaxation after 5 min, where the circumferential and radial relaxations are 5.42 ± 5.94 kPa and 4.5± 3.63 kPa, respectively (*p* = 0.7314). The stress relaxation after 15 min did not produce any statistical difference in the circumferential 10.91 ± 8.30 kPa and radial directions 8.90 ± 8.02 (*p* = 0.6528). To end the stress relaxation test of 30 min, it can be observed that the pericardium stress relaxation in the circumferential direction, 15.28 ± 9.21 kPa, and radial direction 9.6 ± 7.56 kPa (*p* = 0.2308) do not have statistical significance.

## 4. Discussion

### 4.1. Biaxial Tensile Creep

Aortic valves are subjected to sustained stresses under physiological conditions [33]. It is important to understand the behavior of the potential ideal material for the manufacturing of aortic valves. Durable materials are necessary to manufacture durable prosthetic aortic valves; in order to find durability properties of landrace pig’s pericardium, creep tests were performed by subjecting a maintained force of 0.5 N, which is equivalent to 0.25 MPa on the pericardium, so this was used to evaluate time dependent behavior. It was observed that the creep behavior in the circumferential and radial directions was the same after 1 s, 60 s, 300 s, 900 s and 1800 s. The porcine pericardium showed that it is an anisotropic material [39]. The radial direction starting strain under the creep test was more than that of the circumferential direction, even though both directions had statistically the same creep behavior. Under biaxial tensile creep, it was observed that the radial direction had an initial strain starting point which was greater than that in the circumferential direction, which was the same finding for [40,41]. When the creep test started, the change in strain in both directions had no statistical significance, which is similar to the findings by [42]. Generalized Kelvin–Voigt constitutive model fitting was done to find viscoelastic material parameters using the Levenberg–Marquardt algorithm, which are suitable for finite element analysis. The material parameters found in the fitting of the experimental results can be used for finite element analysis and to further synthesize the pericardium, since they fit the experimental data with minimum error, as seen in Table 1 and Table 2 and Figure 6.

The average strain compliance seen after 30 min was less than 1% strain, and from 1 s to 15 min, the maximum strain change was observed. A change in strain of less than 1% assures the material’s ability to maintain its structural integrity and have a longer life service in operation. The applied stress was 250 kPa, which is 10 times greater than the blood pressure of a person with hypertension. The material minimum change in length means that the material would not deform and it is anisotropic; these results promise to produce a durable bioprosthetic aortic valve.

### 4.2. Biaxial Tensile Stress Relaxation

The stress relaxation compliments creep test, the focus of the stress relaxation test, as the name suggests, was to observe how the change in stress differs for the radial and circumferential directions when subjected to a uniform strain of 10% strain. The circumferential direction carries more stress than the radial direction at initial stress relaxation tests, and the change in stress in both directions did not show any statistical significance. As the circumferential direction reduces the amount of stress it could carry at 10% strain, the radial direction did the same, which is like in [25,38,42]. To find viscoelastic material parameters, the generalized Maxwell model was used to fit stress relaxation test results, and as seen from Figure 8, Table 3 and Table 4, the model fitted the experimental data accordingly. Material parameters obtained from curve-fitting by using the generalized Maxwell model can be used for finite element analysis and the development of an idealized prosthetic aortic valve.

Stress relaxation complements creep; when the aortic valve is in operation, it is subjected to sustained forces during systole and diastole, so accessing the material’s ability to hold stress for longer periods was found necessary. To avoid regurgitation, known as backward flow, the aortic valve must be able to apply the force in the same but opposite direction to the blood flowing through it. The results of stress relaxation show relaxation of less than 6.5%, and it is a good sign that the material will do its primary function and completely seal to avoid leakages during diastole and open sufficiently in systole to supply enough blood to the body. The applied constant strain of 10% was possible from an applied stress of 250 kPa, which is not applicable for aortic valves in operation, though the material still maintained 93.5% of the stress, which indicates the durability of the material.

### 4.3. Clinical Significance and Implications

Recent large-scale studies provide robust evidence for the excellent clinical performance of porcine bioprostheses [24]. Porcine valves had a significantly lower rate of reintervention (19% vs. 26% at 20 years) [43]. This advantage was particularly pronounced in larger valve sizes (≥25 mm), leading the authors to suggest porcine valves may be preferable in such scenarios [44].

Structural valve deterioration is the primary reason bioprostheses fail over time [45]. Anselmi et al. demonstrated that porcine valves had significantly lower reoperation rates and better long-term function than pericardial valves in the pulmonic position over a median follow-up of 10.5 years. Nearly 4000 patients were studied. Additionally, a feasibility trial of a new porcine pericardial valve for percutaneous pulmonary implantation showed good short-term effectiveness, indicating continued innovation with this material.

Investigating the viscoelastic properties (like creep and stress relaxation under biaxial loading) is the key to understanding why porcine tissue is more resistant to failure [46].

Additionally, creep and viscoelastic properties can be used in FSI models to simulate decades of wear and tear, helping biomedical engineers optimize valve design and potentially extend their lifespan. FSI models of aortic valve can help to evaluate and analyze the healthy state, pathology, and treatment methods [47,48,49]. Clinical studies comparing porcine and pericardial bioprostheses are summarized in Table 5. Over a 20-year period, the porcine valve demonstrated significantly fewer reinterventions [50]. Additionally, it showed considerably better freedom from structural valve deterioration at the 10-year follow-up [51]. At the 10.5-year follow-up, the porcine valve also had a lower reoperation rate [44]. A new porcine-pericardial valve exhibited favorable short-term results during a six-month follow-up [52]. Biological valves are widely used and have shown good outcomes over a 15-year period [53].

**Table 5:** Key clinical studies on porcine vs. pericardial bioprostheses.

Study Focus	Key Finding on Porcine Valves	Patient Population/Follow-Up	Citation
20-Year Outcomes (Porcine vs. Pericardial)	Lower reintervention rate for porcine valves (19% vs. 26% at 20 years). Equivalent long-term survival.	A total of 1306 SAVR patients. Mean age 68. Twenty-year follow-up.	[50]
Long-Term Performance (Porcine vs. Pericardial)	Significantly better freedom from SVD for porcine valves at 10 years (98.0% vs. 96.3%).	A total of 3983 SAVR patients. Median follow-up of 10.4 years.	[51]
Pulmonic Position Durability (Porcine vs. Pericardial)	Porcine valves had lower reoperation rates and better long-term valve function.	A total of 258 cases (pulmonic position). Mean age 14.9. Median follow-up 10.5 years.	[44]
New Valve Feasibility (α-Gal-Free Porcine Pericardium)	New porcine pericardial valve (Pulsta) showed good short-term effectiveness and safety.	Ten patients (pulmonic position). Six-month follow-up.	[52]
Mechanical vs. Biological (General)	Biological valves, in general, are increasingly used and show good long-term outcomes, avoiding anticoagulation risks.	A total of 45,639 AVR/MVR patients. Up to 15-year follow-up.	[53]

### 4.4. Future Application of Results

The current application of results can be in design of novel TAVI bioprosthesis. TAVI bioprosthesis consists of nitinol stent frame [13,54] and leaflets extracted from animals. When you squash a new valve into a calcified old valve, how much does the tissue creep? If the tissue creeps (slowly deforms) after implantation, the valve cannot seal perfectly against the wall, causing blood to leak around the edges. If the tissue cannot relax the stress, the calcium might crack the new valve. Experimental creep analysis will assist in predicting how the valve will seat itself in the days after surgery. Stress relaxation tests would be able to find out which tissue treatments (anti-calcification treatments) actually allow the tissue to “relax” better, thereby extending the lifespan of the valve. Every time the valve closes, it experiences sudden stress. If the tissue has poor stress relaxation properties, it cannot dissipate that energy.

In some operated patients, blood is not moving as fast as expected across the valve. Perhaps the valve tissue has high viscoelasticity. It opens slowly or incompletely due to the material stiffness, not just the geometric narrowing. To correlate creep deformation rates with patient symptoms, it would help to evaluate a valve because it deforms poorly under load, even if the opening looks moderate.

The data obtained during such tests can be employed in numerical FSI simulations to analyze seal of the prosthesis, paravalvular leakage and low-pressure gradients mentioned above.

## 5. Conclusions

Aortic valves are expected to last for a long time, and the effective design of prosthetic aortic valves, which involves durable viscoelastic material parameters, is of importance. Changes in the circumferential direction and the radial direction in strain when subjected to stress are approximately the same, even though the radial direction carries more strain. Future studies should focus on porcine pericardium experimental tests applications to enhance the material for designing and optimized aortic valve and preclinical studies to assess the material performance in vivo. The results showed that the creep behavior was the same in both directions after 1 s, 60 s, 300 s, 900 s, and 1800 s. After 30 min of creep, deformation in the circumferential and radial directions was 3303 $\times \;10^{-6}$ and 5192.9 $\times \;10^{-6}$, respectively. Stress relaxation tests showed the same behavior as creep. At the stress relaxation test after 30 min, the pericardium deformation in the circumferential and radial directions was 15.28 kPa and 9.6 kPa, respectively. The Prony series with Levenberg–Marquardt as the optimizer was used to obtain material parameters to use for finite element analysis. The data obtained during such tests can be employed in numerical FSI simulations of novel aortic valve bioprosthesis long-term performance in a patient’s body.

## Figures and Tables

**Table 1:** Circumferential direction parameters from fitting the generalized Kelvin–Voigt model to experimental data.

	Creep_1	Creep_2	Creep_3	Creep_4	Creep_5	Creep_6	Creep_7	Average
$J_{0}$	2 × 10^3^	1 × 10^3^	2 × 10^3^	2 × 10^3^	1 × 10^3^	7 × 10^2^	1 × 10^3^	1 × 10^3^
$J_{1}$	78.13	83.95	79.54	63.40	59.51	27.88	45.08	65.40
$\tau_{1}$	2.01	2.36	0.56	0.61	2.98	3.18	0.78	1.95
$J_{2}$	0.00	0.00	3.06	1.37	0.00	1.09	0.13	0.92
$\tau_{2}$	80.56	97.25	12.22	57.79	476.73	152.74	40.63	146.22
$J_{3}$	0.02	0.11	5.79	12.92	4.73	49.94	4.82	12.25
$\tau_{3}$	2 × 10^5^	1 × 10^5^	2 × 10^3^	3 × 10^3^	9 × 10^2^	2 × 10^4^	3 × 10^3^	5 × 10^4^
R2	0.94	0.90	0.97	0.97	0.99	1.00	0.96	0.96
r	0.97	0.95	0.98	0.99	1.00	1.00	0.98	0.98
ASE	3 × 10^−3^	9 × 10^−3^	2 × 10^−4^	1 × 10^−3^	2 × 10^−4^	3 × 10^−4^	3 × 10^−4^	2 × 10^−3^
NRMSE	0.01	0.01	0.04	0.04	0.03	0.01	0.07	0.03

**Table 2:** Radial direction parameters from fitting the generalized Kelvin–Voigt model to experimental data.

	Creep_1	Creep_2	Creep_3	Creep_4	Creep_5	Creep_6	Creep_7	Average
$J_{0}$	9 × 10^2^	8 × 10^2^	7 × 10^2^	8 × 10^2^	8 × 10^3^	1 × 10^3^	8 × 10^2^	2 × 10^3^
$J_{1}$	123.88	63.64	50.37	58.20	467.10	51.61	42.88	122.53
$\tau_{1}$	5.00	7.67	0.12	3.29	47.34	0.46	4.40	9.76
$J_{2}$	2.12	2.26	0.74	0.72	0.01	3.06	1.02	1.42
$\tau_{2}$	1 × 10^2^	9 × 10^1^	8 × 10^1^	3 × 10^1^	2 × 10^3^	2 × 10^1^	2 × 10^2^	4 × 10^2^
$J_{3}$	9.90	10.19	5.05	4.26	29.33	7.87	3.36	9.99
$\tau_{3}$	4 × 10^3^	3 × 10^3^	8 × 10^3^	1 × 10^3^	1 × 10^4^	4 × 10^3^	2 × 10^3^	5 × 10^3^
R2	0.99	0.99	0.98	0.99	0.90	0.99	0.99	0.98
r	1.00	1.00	0.99	0.99	0.95	0.99	0.99	0.99
ASE	4 × 10^−4^	5 × 10^−4^	1 × 10^−4^	3 × 10^−5^	2 × 10^−3^	3 × 10^−4^	3 × 10^−4^	5 × 10^−4^
NRMSE	0.01	0.01	0.03	0.03	0.07	0.03	0.02	0.03

**Table 3:** Circumferential direction parameters from fitting the generalized Maxwell model to experimental data.

	Rela_1	Rela_2	Rela_3	Rela_4	Rela_5	Rela_6	Rela_7	Average
$\sigma_{0}$	0.22	0.02	0.00	0.01	0.00	0.09	0.09	0.06
$\sigma_{1}$	4 × 10^−3^	3 × 10^−4^	4 × 10^−2^	2 × 10^−1^	5 × 10^−3^	1 × 10^−4^	1 × 10^−3^	4 × 10^−2^
$\tau_{1}$	0.41	0.32	28.64	0.82	5.54	0.04	1.28	5.29
$\sigma_{2}$	0.04	0.00	0.00	0.07	0.01	0.00	0.33	0.06
$\tau_{2}$	66.44	615.13	33.01	8.79	12.87	14.89	3.62	107.82
$\sigma_{3}$	0.03	0.01	0.10	0.06	0.13	0.01	0.04	0.06
$\tau_{3}$	1 × 10^3^	9 × 10^2^	2 × 10^4^	2 × 10^4^	1 × 10^4^	5 × 10^2^	1 × 10^3^	7 × 10^3^
R2	0.94	0.89	0.93	0.88	0.77	0.93	0.91	0.89
r	0.97	0.94	0.97	0.94	0.88	0.96	0.95	0.94
ASE	0.13	0.02	0.03	0.01	0.17	0.03	0.29	0.10
NRMSE	0.05	0.08	0.03	0.04	0.08	0.07	0.09	0.06

**Table 4:** Radial direction parameters from fitting the generalized Maxwell model to experimental data.

	Rela_1	Rela_2	Rela_3	Rela_4	Rela_5	Rela_6	Rela_7	Average
$\sigma_{0}$	0.08	0.00	0.08	0.03	0.00	0.01	0.01	0.03
$\sigma_{1}$	0.00	8 × 10^−2^	4 × 10^−6^	1 × 10^−3^	2 × 10^−3^	2 × 10^−1^	6 × 10^−3^	4 × 10^−2^
$\tau_{1}$	5.60	12.16	0.02	10.53	0.48	6.36	1.17	5.19
$\sigma_{2}$	1 × 10^−2^	3 × 10^−3^	0.E+00	3 × 10^−4^	0.00	9 × 10^−3^	1 × 10^−2^	5 × 10^−3^
$\tau_{2}$	108.00	14.43	517.19	60.23	5.76	42.74	541.09	184.20
$\sigma_{3}$	0.01	0.09	0.03	0.00	0.10	0.06	0.01	0.04
$\tau_{3}$	6 × 10^4^	3 × 10^4^	8 × 10^2^	3 × 10^4^	5 × 10^3^	4 × 10^4^	6 × 10^2^	2 × 10^4^
R2	0.87	0.83	0.95	0.23	0.89	0.72	0.79	0.75
r	0.94	0.91	0.97	0.48	0.94	0.85	0.89	0.85
ASE	0.01	0.02	0.11	0.00	0.27	0.02	0.14	0.08
NRMSE	0.08	0.03	0.06	0.06	0.11	0.04	0.09	0.07

## Data Availability

The data presented in this study are available on request from the corresponding author due to (ethical reasons).
